# Altered synaptic connectivity in an *in vitro* human model of STXBP1 encephalopathy

**DOI:** 10.1093/brain/awac396

**Published:** 2022-10-31

**Authors:** Faye McLeod, Anna Dimtsi, Amy C Marshall, David Lewis-Smith, Rhys Thomas, Gavin J Clowry, Andrew J Trevelyan

**Affiliations:** Biosciences Institute, Newcastle University, Newcastle upon Tyne NE2 4HH, UK; Biosciences Institute, Newcastle University, Newcastle upon Tyne NE2 4HH, UK; Biosciences Institute, Newcastle University, Newcastle upon Tyne NE2 4HH, UK; Newcastle University Translational and Clinical Research Institute, Newcastle upon Tyne NE2 4HH, UK; Department of Clinical Neurosciences, Royal Victoria Infirmary, Newcastle Upon Tyne Hospitals NHS Foundation Trust, Newcastle upon Tyne NE1 4LP, UK; Newcastle University Translational and Clinical Research Institute, Newcastle upon Tyne NE2 4HH, UK; Department of Clinical Neurosciences, Royal Victoria Infirmary, Newcastle Upon Tyne Hospitals NHS Foundation Trust, Newcastle upon Tyne NE1 4LP, UK; Biosciences Institute, Newcastle University, Newcastle upon Tyne NE2 4HH, UK; Biosciences Institute, Newcastle University, Newcastle upon Tyne NE2 4HH, UK

**Keywords:** human organotypic slices, synapses, STXBP1, subplate, neurodevelopment

## Abstract

Early infantile developmental and epileptic encephalopathies are devastating conditions, generally of genetic origin, but the pathological mechanisms often remain obscure. A major obstacle in this field of research is the difficulty of studying cortical brain development in humans, at the relevant time period *in utero*. To address this, we established an *in vitro* assay to study the impact of gene variants on the developing human brain by using living organotypic cultures of the human subplate and neighbouring cortical regions, prepared from ethically sourced, 14–17 post-conception week brain tissue (www.hdbr.org). We were able to maintain cultures for several months, during which time the gross anatomical structures of the cortical plate, subplate and marginal zone persisted, while neurons continued to develop morphologically and form new synaptic networks. This preparation thus permits the study of genetic manipulations and their downstream effects on an intact developing human cortical network. We focused on STXBP1 haploinsufficiency, which is among the most common genetic causes of developmental and epileptic encephalopathy. This was induced using shRNA interference, leading to impaired synaptic function and a reduced density of glutamatergic synapses. We thereby provide a critical proof-of-principle for how to study the impact of any gene of interest on the development of the human cortex.

## Teaser

The pathology of genetic epilepsies is likely to begin *in utero*. McLeod *et al.* establish an *in vitro* assay to study the impact of specific gene variants on the developing human brain, using living organotypic slice cultures of the early human cortex, prepared from ethically sourced tissue samples.

## Introduction

Approximately 1 in 2000 children in the UK develop epilepsy before 3 years of age from a monogenic cause^1^; of these, about a third will continue to have seizures into adulthood.^2^ Knowing the genetic cause aids clinical management, yet the true pathological mechanisms remain unknown, at least in part because the aetiology often starts *in utero*, making their study extremely difficult. A critical phase of prenatal neocortical development is the transient appearance, and subsequent dissolution, of the subplate. This cortical region contains the earliest cortical synaptic network and forms the scaffold for subsequent neocortical development.^3,4^ It is therefore the site where synaptic malfunction will first disrupt the developing cortex.

Of particular interest is Syntaxin-binding protein 1 (STXBP1), an essential pre-synaptic protein also called MUNC18-1 (Mammalian uncoordinated-18),^5^ that is highly expressed in the prenatal developing brain.^6^ Loss of function variants in *STXBP1*, collectively termed ‘STXBP1 encephalopathies’, result in significant intellectual disability without regression, early-life onset epilepsy in most, and a range of movement disorders including ataxia, tremor and spasticity,^7–9^ but the underlying pathological mechanisms are poorly understood. Individuals with protein truncating variants seem to have similar clinical presentations when compared in a large cohort with STXBP1 encephalopathies, suggesting a shared pathological aetiology.^10^ It seems likely that the effects of STXBP1 haploinsufficiency arise prenatally, supported by the observation that STXBP1 is highly expressed in the developing subplate.^6^ The study of functional prenatal interactions, however, presents a major practical challenge for early human development.

STXBP1 haploinsufficiency has been replicated in transgenic mouse models and is associated with a post-natal deficit in synaptic neurotransmission.^11,12^ Rodent models of this condition, however, are limited in one important respect: the rodent subplate is small and very simple in organization, compared with the human subplate,^13^ calling into question the relevance of these rodent models to human development. Likewise, human cerebral organoids are similarly limited, because, to date, there have been no descriptions of any organoid structures that accurately resemble the subplate as it exists *in utero.*^14^

To overcome this critical gap in our experimental arsenal, we have developed a protocol for preparing living organotypic cultures of human neocortex, and its underlying subplate, from ethically approved samples of human foetal brain tissue, provided by the MRC-Wellcome Trust Human Developmental Biology Resource (HDBR, www.hdbr.org). Over time, neurons within the culture display evolving morphology, while the culture itself retains its gross anatomical structure. Importantly, the cultures are amenable to genetic manipulation, including knock-down of STXBP1. This is achieved using short hairpin RNA (shRNA) interference, delivered by adeno-associated virus (AAV) viral vectors, to mimic the disease-causing, haploinsufficient state, and by using viral vectors carrying a scrambled *STXBP1* sequence as a control. STXBP1 knock-down results in altered synaptic stability and changes in the number of synaptic terminals. We have thus developed a model that is closer to early human neurodevelopment than existing alternatives, and able to facilitate the study of mechanisms suspected of causing neurodevelopmental disorders in humans.

## Materials and methods

Detailed methods are provided in the Supplementary material.

### Human organotypic slice cultures

Human foetal brain tissue was obtained from the joint MRC/Wellcome Trust funded HDBR (https://www.hdbr.org/Project 200428). All tissue was collected with appropriate maternal consent. Ethical approval was granted from the Newcastle and North Tyneside NHS Health Authority Joint Ethics Committee (REC reference: 18/NE/0290). Cultures were prepared from 10 different foetal brains [three from 14 post-conception weeks (pcw), four 16 pcw and three 17 pcw]. Slices were sectioned on a vibratome (280 µm), and then transferred to six-transwell plates containing culture media, which was changed every 2 days. The plates were maintained at 37°C, 5% CO_2_ in ambient O_2_ and 90% humidity.

### Viral vectors

Three AAV vectors were used:

- Scrambled control shRNA—packaged into an AAV9 vector expressing the cytomegalovirus (CMV) promoter and enhanced green fluorescent protein (eGFP) to achieve a final titre of 5 × 10^12^ GC/ml. The scrambled target sequence was 5′-CCTAAGGTTAAGTCGCCCTCG-3′. Infected cells were referred to as ‘control’. This virus was purchased from VectorBuilder (VB010000-0023jze).
- STXBP1 shRNA—packaged into an AAV9 vector expressing the cytomegalovirus promoter and eGFP to achieve a final titre of 5 × 10^12^ GC/ml. The STXBP1 target sequence was 5′TACTGAAGCACAAACATATAT-3′. This virus was purchased from VectorBuilder (VB210925-1040cqu).
- AAV9-CMV-GFP (final titre of 1 × 10^13^ GC/ml). This virus was made in-house.

Infected cells were identified by their eGFP/GFP tag. At 0 days *in vitro* (DIV), 3–5 μl of viral vectors were added to the top of each slice. Experiments were conducted up to 4 weeks later for neurite time-lapse experiments, and 2 weeks later for all genetic knock-down conditions.

### Live imaging

Live imaging was performed using an inverted Zeiss LSM800 Airyscan confocal microscope. FM4-64 dye was used as previously described^15^ and analysis was performed using Fiji software (NIH, RRID:SCR_002285).

### Whole cell-patch clamp recordings

Slices were transferred into the recording chamber of an upright Leica DMLFSA fluorescent microscope fitted with Micro Control Instruments micromanipulators, and continuously perfused at 34°C with oxygenated artificial CSF. AMPA receptor-mediated miniature glutamatergic post-synaptic currents were recorded at −60 mV in the presence of 100 nM TTX, 10 μM bicuculline and 50 μM AP-5. Miniature GABAergic post-synaptic currents were recorded at 0 mV in the presence of 100 nM TTX and 50 μM AP-5.

### Immunohistochemistry

Staining was performed using a protocol optimized specifically for thick slices.^16^ All primary antibodies used in this study are documented in Supplementary Table 2.

### Image acquisition and analysis

Images were acquired on a Zeiss LSM800 Airyscan confocal microscope with fixed settings. The 5× objective [numerical aperture (NA) = 0.16] was used to acquire image stacks (*z*-step = 5 μm) of general slice anatomy. The 10× objective was used to aid identification of the subplate. This region has an easily identifiable morphology with a sparse cell population in comparison to the neighbouring cortical plate. Subsequently, the 20× objective (NA = 0.8) with ×2 zoom was used to acquire image stacks (z-step = 2 μm) of the infected and non-infected subplate neurons and the 63× objective (NA = 1.4) to acquire image stacks (*z*-step = 0.25 μm) of synaptic components. Three to four image stacks in the upper part of the subplate (neighbouring the cortical plate) were acquired per slice in a randomized manner. A final image resolution of 1024 × 1024 pixels was obtained.

The number as well as the length of primary subplate neuron processes for scrambled shRNA control and STXBP1 shRNA conditions were measured using Fiji software. Synaptic counts and integrated density (area × mean intensity) measurements were conducted using Zen v.3.1 software (Zeiss; RRID:SCR_013672).

### Western blot

Equal homogenate amounts were run on 10% sodium dodecyl–sulphate polyacrylamide gel electrophoresis gel and transferred onto polyvinyl difluoride membranes using BIO-RAD western blot apparatus.

### Statistical analysis

Statistical analyses were performed on GraphPad Prism v.9 (RRID:SCR_002798). We assessed data normality using Kolmogorov–Smirnov tests. The appropriate parametric test was used for all normally distributed data, including paired/unpaired *t*-tests, one-way ANOVA and two-way ANOVA with Tukey’s multiple comparisons test and repeated measures. Statistical significance was accepted as *P < 0.05, **P < 0.01 and ***P < 0.001, and non-significant data are indicated as ns. All data in the results section are presented as ±SEM. Further details of exact sample replicates and statistical tests used are stated in the ‘Results’ section and each figure legend.

### Data availability

All data generated or analysed and used in this study are available upon reasonable request.

## Results

### STXBP1 expression in the developing human brain

**Figure 1:**
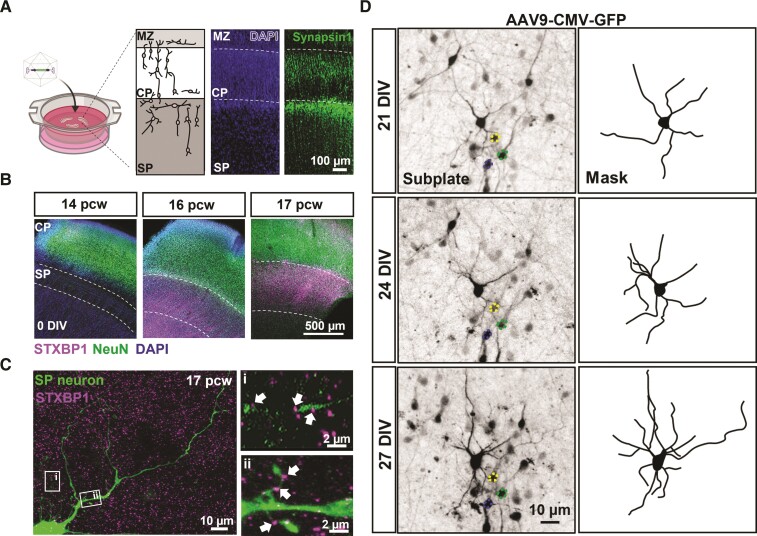
**Human organotypic cortical brain slices cultures with developing subplate neurons.** (**A**) Schematic and low magnification photomicrographs of organotypic cultures prepared from human foetal brain tissue (created with BioRender.com). Cultures are maintained for 1–4 weeks following infection with and without different viral vectors. Micrograph image represents a 16 pcw sample, 2 weeks in culture with clearly identifiable cortical regions including the marginal zone (MZ), cortical plate (CP) and subplate (SP). DAPI labels all cell nuclei and Synapsin1 immunohistochemistry labels reveals pre-synaptic terminals predominantly located in SP but also CP and MZ. (**B**) Representative images from 14, 16 and 17 pcw cultures (0 DIV). STXBP1 and NeuN (all neurons) immunofluorescence reveals location of the SP. Note the enrichment of STXBP1 within the SP at 16–17 pcw (*n* = 3 human samples/age). (**C**) SP neuron (eGFP transduced cell) with STXBP1 present on neurites along thin putative axons extending from the cell body [**C**(**i**)] and spine-like structures [**C**(**ii**)] indicated by the arrows. (**D**) Photomicrographs of a 16 pcw culture infected with AAV9-CMV-GFP and taken at 7, 21 and 27 DIV, showing the same neuron (highlighted by the masked images). Note the dynamic nature and elaboration of neurites over time. Coloured outlines highlight reference cells that are stable overtime.

We prepared organotypic slice cultures of occipital neocortex and the underlying subplate, from fresh human foetal brain tissue samples from 12 different individuals (Supplementary Table 1). At the time of preparation, the *in utero* developmental age of the samples was 14–17 pcw. In all cases, the cultures retained the distinct and identifiable anatomical regions of the subplate, its neighbouring cortical plate and the marginal zone (Fig. 1A and B). Synapsin1 immunoreactivity revealed that pre-synaptic terminals were predominantly located in the upper layer of the subplate (adjacent to the cortical plate) at this stage of development (Fig. 1A). The subplate was smallest in 14 pcw samples (*n* = 3 individual samples; average depth = 308 ± 57 µm), becoming progressively larger in the cultures prepared from 16 pcw (*n* = 4; 525 ± 11 µm) and 17 pcw brains (*n* = 3; 651 ± 33 µm) (*P* < 0.05, one-way ANOVA; Fig. 1B). Similarly, the cortical plate also increased in size from 14 pcw (average depth = 355 ± 26 µm; *n* = 3) to 17 pcw (477 ± 54 µm; *n* = 3) (*P* < 0.05, unpaired *t*-test; Fig. 1B). STXBP1 showed higher expression at 16–17 pcw, compared to 14 pcw (Fig. 1B), and was observed near spine-like structures and along putative axons of subplate neurons (Fig. 1D). Collectively, these data show that the subplate becomes more prominent and enriched with pre-synaptic proteins including STXBP1 over the 14–17 pcw period.

### Synapse maturation in human cortical slice cultures

Given the relatively higher expression of STXBP1 in the subplate at 16–17 pcw and higher density of pre-synaptic terminals in the upper subplate (Fig. 1A), we focused on this age and location for subsequent analysis of how the organotypic cultures continue to develop *in vitro.* To examine how neuronal morphology developed in the human cortical slice cultures, we infected cultures with viral vectors carrying the eGFP construct. We were able to resolve the neurites of individual labelled neurons and follow their development over intervals of many days to weeks, *in vitro*. Over these time periods, there was a marked elaboration of the neurite processes (Fig. 1D).

**Figure 2:**
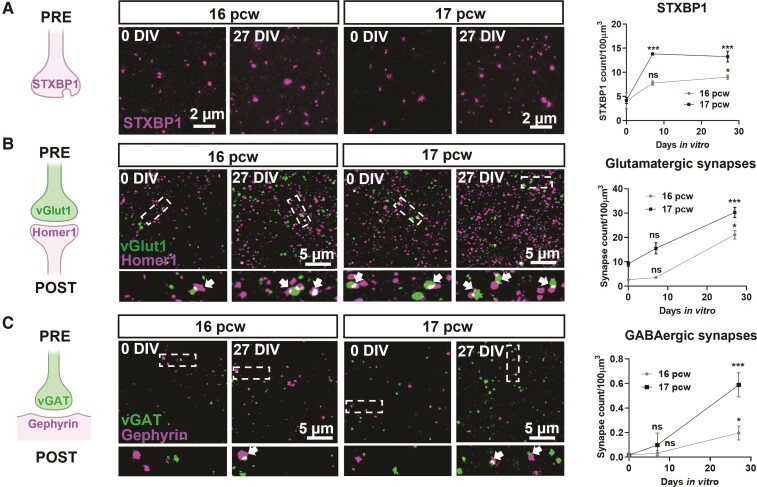
**Synapse maturation in human cortical slice cultures.** (**A**) *Left* shows the schematic displaying the pre-synaptic location of STXBP1. *Middle* shows the photomicrographs of STXBP1 in the subplate of 16–17 pcw cultures at 0 and 27 DIV. *Right* shows the quantification of the increase in the number of STXBP1 puncta over time at both ages. (**B**) *Left to middle* shows the colocalization of the pre- and post-synaptic glutamatergic markers, vGlut1 and Homer1, respectively indicated by arrows, in the subplate at 0 and 27 DIV. Areas, demarcated by dotted boxes in the upper micrographs, are shown enlarged below. All cultures were prepared at 16–17 pcw. (**C**) Similar analyses performed for GABAergic synapses based on colocalization of vGAT (pre-synaptic) and Gephyrin (post-synaptic). Analyses for **A**–**C** were performed as two-way ANOVA with repeated measures from comparisons made at 0 DIV for each age, *n* = 6–8 slices per time point from 2–3 human samples/age. Error bars indicate SEM.

The number of STXBP1 immunoreactive puncta in the subplate more than doubled, between 0 and 27 DIV (16 pcw cultures increase from 4.1 ± 1.6 to 9.0 ± 0.4 puncta/100 µm^3^; 17 pcw cultures increase from 4.2 ± 0.6 to 13.3 ± 1.1 puncta/100 µm^3^; Fig. 2A). STXBP1 is localized within glutamatergic and GABAergic pre-synaptic components (Supplementary Fig. 1). We also found significant increases in both glutamatergic synapses (vGlut1 on Homer1 colocalization; 16 pcw cultures increase from 2.7 ± 0.5 to 21.1 ± 1.7 puncta/100 µm^3^; 17 pcw cultures increase from 9.2 ± 0.9 to 30.3 ± 2.1 puncta/100 µm^3^; Fig. 2B), and GABAergic synapses (vGAT on gephyrin colocalization; 16 pcw, increase from 0.02 ± 0.02 to 0.2 ± 0.01 puncta/100 µm^3^; 17 pcw, increase from 0.02 ± 0.02 to 0.5 ± 0.05 puncta/100 µm^3^; Fig. 2C). These data show that in the human organotypic cultures, subplate neurons continue to mature, making both glutamatergic and GABAergic synapses.

### Divergent regulation of synapses with reduced STXBP1 levels

**Figure 3:**
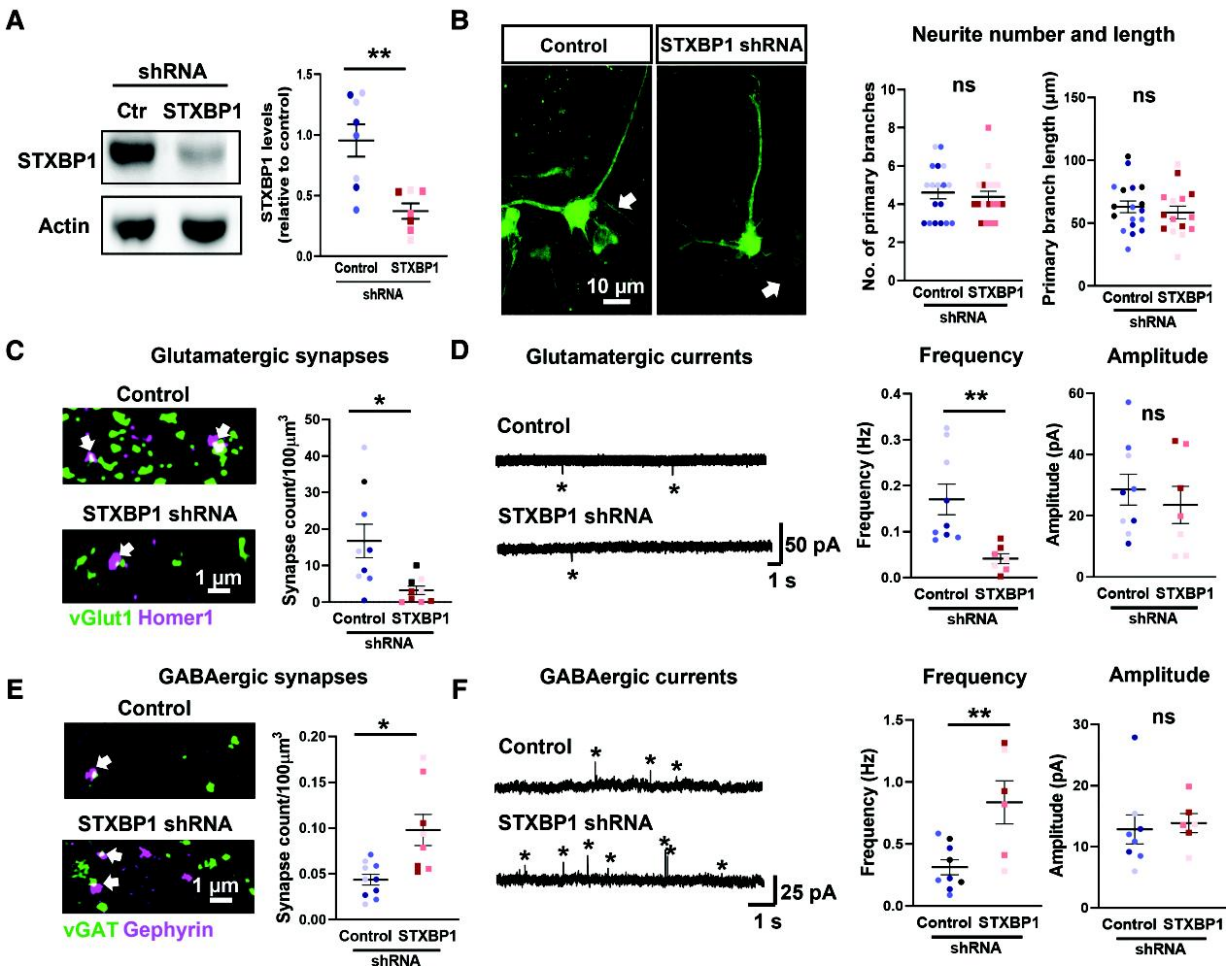
**Divergent regulation of glutamatergic and GABAergic synapses in the subplate following knock-down of STXBP1.** (**A**) *Left* shows a western blot showing knock-down of STXBP1 protein in a whole slice at 16 pcw following 14 DIV with scrambled control (Ctr) or *STXBP1* shRNA. *Right* shows a quantification of band intensity showing a ∼50% reduction in STXBP1 levels (unpaired *t*-test, taken from *n* = 8 slices/condition from three human samples). (**B**) *Left* shows the example subplate neurons infected with scrambled control or *STXBP1* shRNA for 14 DIV at 16 pcw. Note the putative axons highlighted with the arrows. *Right* shows the quantification of primary neurite number and length reveals no gross morphology changes in both conditions (*n* = 30 cells from 15–18 slices and three human samples). (**C** and **E**) Quantification of glutamatergic synapses (arrows) (**C**) and GABAergic (**E**) synapses (arrows) in the subplate at 16 pcw following 2 weeks of scrambled control or *STXBP1* shRNA by colocalization of pre- and post-synaptic markers. Analysis performed using an unpaired *t*-test, *n* = 8–10 slices per condition from three human samples. (**D** and **F**) Representative miniature glutamatergic post-synaptic currents recorded at −60 mV (**D**) and miniature GABAergic post-synaptic currents recorded at 0 mV (**F**) from 16 pcw subplate cells in both genetic conditions. The frequency and amplitude of events (starred) were quantified (unpaired *t*-test, *n* = 12 cells and 7–9 slices per condition from three human samples). Data presented as scatter plots and mean ± SEM displaying all slice data-points with each human sample per condition represented by a different colour shade.

We next investigated the effects of manipulating the expression of STXBP1 in the human cortical slice cultures. We created a generic haplo-insufficiency phenotype for STXBP1 (‘knock-down’) in our human cortical slice cultures using an AAV packaged *STXBP1* shRNA in 16 pcw tissue samples, derived from a minimum of three individuals (Fig. 3 and Supplementary Fig. 2). To control for any effects of the viral infection, we performed similar infections with a scrambled STXBP1 sequence, in age matched brain slices (‘scrambled control’). The shRNA achieved ∼50% downregulation of STXBP1 at 14 DIV, compared with the scrambled control cultures prepared from the same brain tissue sample (Fig. 3A). Within the subplate, STXBP1 immunoreactivity was reduced by ∼30%, although there was no overall change in the absolute number of STXBP1 puncta (Supplementary Fig. 3). Since each punctum was presumed to represent a synaptic terminal, this suggests that our shRNA design reduces endogenous STXBP1 protein levels without affecting the total number of STXBP1-positive synaptic terminals.

At 14 DIV, we found no difference in the size of the subplate between control (*n* = 10 slices, average depth = 529 ± 25 µm) and knock-down cultures (*n* = 10, average depth = 549 ± 21 µm; *P* > 0.05, unpaired test). Furthermore, the number and length of neurite processes in subplate neurons in STXBP1 knock-down cultures was indistinguishable (Fig. 3B). In contrast, in these same knock-down cultures, there was a 51% drop in the pre-synaptic vGlut1 puncta (control = 56.8 ± 7.2 puncta/100 µm^3^; STXBP1 knock-down = 27.8 ± 5.4 puncta/100 µm^3^) and a 48% drop in the post-synaptic Homer1 puncta (control = 45.8 ± 4.1 puncta/100 µm^3^; STXBP1 knock-down = 23.6 ± 5.6 puncta/100 µm^3^) within the subplate region (Supplementary Fig. 4A). We next asked whether there was a concomitant change in the number of co-localized vGlut1 and Homer1 puncta, indicative of full synaptic contact. If the changes in these two proteins occur independently, then one would expect a ∼75% drop in the co-localized proteins; we found an 80% drop (normalized to the control average; 95% confidence intervals = 67–94%), which matched this predicted drop closely (control = 16.7 ± 4.5 puncta/100 µm^3^; STXBP1 knock-down = 3.3 ± 3.2 puncta/100 µm^3^; Fig. 3C). To evaluate the functional effects of these anatomical changes, we recorded glutamatergic currents at 14 DIV. The frequency of synaptic events was decreased by 75% in cells expressing *STXBP1* shRNA (Fig. 3D).

Remarkably, the genetic manipulation had the opposite effect on GABAergic synapses in cultures undergoing STXBP1 knock-down. There was minimal change in the individual synaptic components (pre-synaptic vGAT: control, 8.4 ± 1.4 puncta/100 µm^3^, STXBP1 knock-down, 7.2 ± 1.3 puncta/100 µm^3^; post-synaptic gephyrin: control, 1.07 ± 0.1 puncta/100 µm^3^, STXBP1 knock-down, 1.3 ± 0.1 puncta/100 µm^3^; Supplementary Fig. 4B), but there was a large increase in the colocalization of the pre- and post-synaptic expression, indicating there was a doubling of the number of GABAergic synapses in the STXBP1 knock-down cultures (0.09 ± 0.02 puncta/100 µm^3^) compared with the control cultures (0.04 ± 0.01 puncta/100 µm^3^; Fig. 3E). There was a parallel ∼1.5-fold increase in the frequency of GABAergic post-synaptic currents (Fig. 3F), compared to scrambled control. There was no change, however, in the amplitude of either glutamatergic or GABAergic currents (Fig. 3D and F). Overall, it seems that reducing STXBP1 expression has no effect on the overall stability of primary subplate neuron neurites but does alter synaptic stability during early subplate development.

### Impaired vesicle exocytosis with loss of STXBP1

**Figure 4:**
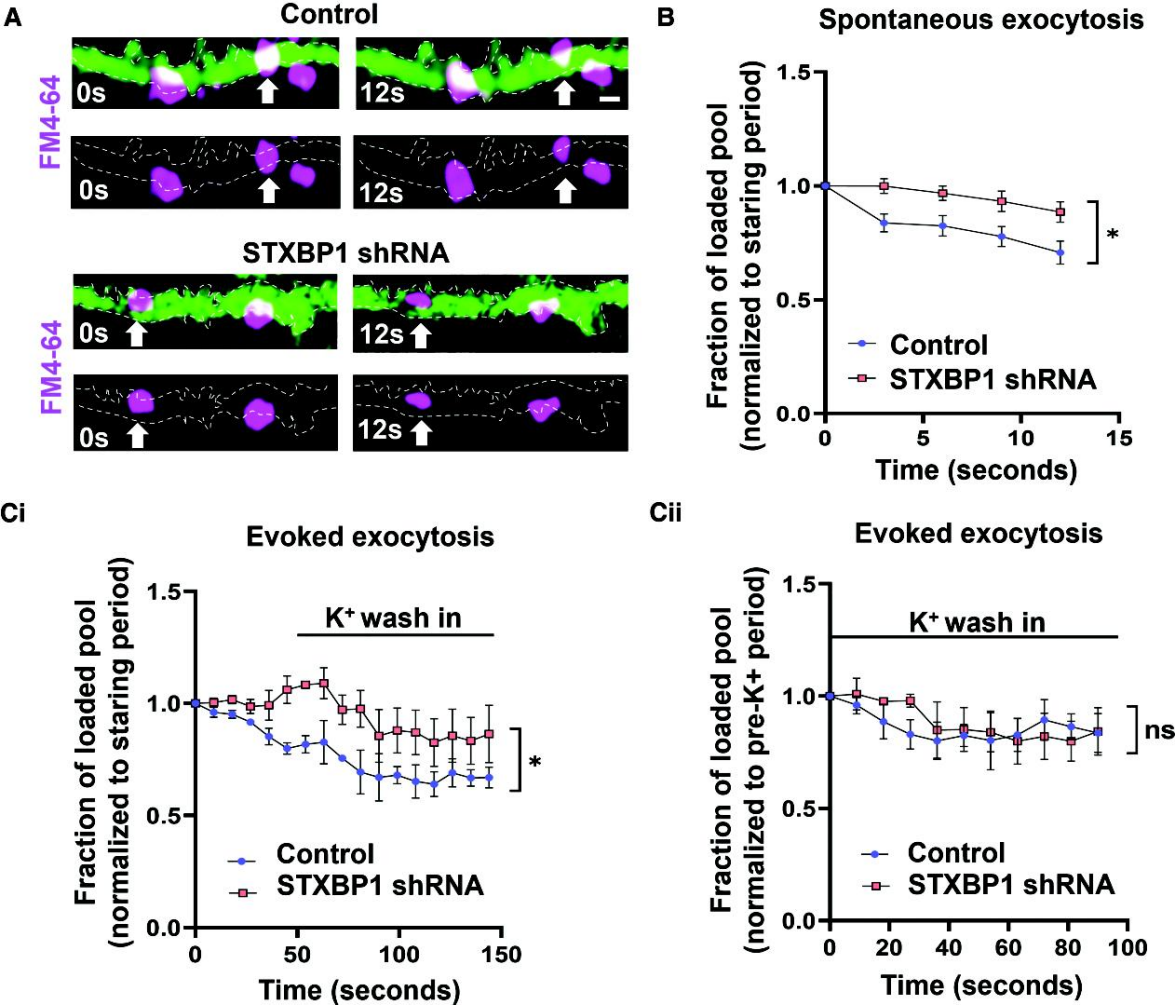
**Impaired vesicle exocytosis with reduced STXBP1 levels.** (**A**) Isolated axons (green) tracked from FM4-64 loaded subplate neurons in scrambled control and STXBP1 shRNA slices (16 pcw) at 14 DIV. Note the presence of spontaneous exocytosis from vesicles loaded with FM4-64 (arrows). Scale bar = 1 µm. (**B**) Quantification of spontaneous exocytosis (*n* = 12 slices from four human samples) in both genetic conditions (without stimulation). (**C**) Quantification of evoked exocytosis in separate slices (*n* = 8 slices from three human samples) following brief potassium chloride (K^+^) stimulation. Data were normalized to the first five frames of the recording (starting period) [**C**(**i**)] or to the five frames immediately prior to K^+^ wash in (pre-K^+^ period) [**C**(**ii**)]. All analysis was performed using a two-way ANOVA with repeated measures. All error bars represented as SEM.

STXBP1 plays a critical role in synaptic vesicle recycling.^17,18^ To assess this function, we assayed depletion of fluorescence in tissue that had been loaded with the lipophilic dye, FM4-64. Synaptic vesicles loaded with FM4-64 were identified along isolated axons in the subplate in 16 pcw tissue (Fig. 4A). At 14 DIV, under baseline conditions, signal intensity was reduced by 30% from 0 to 12 s in scrambled controls but not after STXBP1 shRNA knock-down (Fig. 4B), indicating an impairment in spontaneous FM dye unloading at synapses with reduced STXBP1 levels.

In a separate set of brain slices, we assessed evoked exocytosis, by challenging the slices with 50 mM potassium ions, to induce a maximal level of depolarization-induced vesicle exocytosis. The intensity of the signal started to decrease in the first 60 s in scrambled controls but not after STXBP1 shRNA knock-down (Fig. 4C). On K^+^ stimulation, a 20% reduction in signal intensity was observed in both experimental groups when compared to their pre-evoked levels (Fig. 4C). Given that the magnitude change is the same, this suggests that reduced STXBP1 does not affect evoked FM dye unloading in the subplate under these experimental conditions.

## Discussion

We present a novel means of investigating the effect of genetic disorders on prenatal human cortical development that benefits from retaining subplate and cortical plate neuronal networks. This represents a major advance over other approaches, such as human cerebral organoids or induced pluripotent stem cell cultures, neither of which exhibit synaptic circuitry that approaches the complexity of the real *in utero* networks. In these initial proof-of-principle studies, using a simple shRNA interference genetic manipulation, we demonstrate the divergent effects of STXBP1 haploinsufficiency on glutamatergic and GABAergic synapses at an early stage of brain development. This work will help improve our understanding of the pathological mechanisms underlying certain early infantile onset developmental and epileptic encephalopathies.^11^

Reduced levels of STXBP1 did not affect the gross morphology of subplate neurons at early developmental stages. Previous studies have shown that absence of *UNC-18*, the ortholog gene of *STXBP1*, does not affect neurite length or maintenance in *Caenorhabditis elegans*^18^ and that heterozygous Stxbp1 haploinsufficiency does not affect dendrite length^17^ or cortical neuron density^11^ in mice postnatally. By addressing these questions in a model closer to the developing human cortex, our data provide confirmatory evidence that these findings are relevant also to the human disorder.

Deficiencies in STXBP1 can result in glutamatergic synaptic neurotransmission dysfunction in rodent models^11^ and neurons derived from human embryonic stem cells.^19^ Similarly, we find reduced glutamatergic post-synaptic current frequency, synapse number (equal pre- and post-synaptic component loss) in the human subplate following knock-down of STXBP1. Interestingly, we observed the opposite effect on GABAergic synapses, with an increase both in the frequency of GABAergic synaptic events, and the density of GABAergic synapses. However, there was no change in the absolute number of individual GABAergic pre- and post-synaptic components, suggesting that the coordination of the pre- to post-synaptic elements, to make new synapses, is promoted in the knock-down cultures. STXBP1 is normally present at both GABAergic and glutamatergic synapses, so the divergent effects of STXBP1 knock-down on the two types of synapse was unexpected; given that the two synaptic classes are likely to be regulated in parallel, this divergence may represent a compensatory change manifest in one class, responding to a primary effect in the other. This new finding demonstrates an advantage of our model over previous single cell assays that may not be sensitive to such divergent effects present in an intact neuronal network.

The decrease in frequency of glutamatergic currents and spontaneous FM dye unloading could reflect a decrease in glutamatergic synapses, an impairment in the probability of pre-synaptic neurotransmitter release or, alternatively, a reduction in network excitability. We are unable to distinguish between these possibilities from our experiments. Nonetheless, it is pertinent that our immunohistochemistry data indicate that there is almost an order of magnitude more glutamatergic than GABAergic synapses in the subplate (which is not reflected in the post-synaptic current frequency possibly due to the functional immaturity of the circuit or receptor composition), suggesting that the FM dye labelling is likely to be predominantly of glutamatergic synapses.

Both spontaneous, and evoked neurotransmitter release is affected by lowering the levels of STXBP1 in many animal models.^17–20^ These results are consistent with the observation that at functioning synapses STXBP1 is crucial for the assembly of the SNARE complex, a collection of proteins involved in the fusion of vesicles with the plasma membrane,^21–23^ variants in several of which are implicated in neurodevelopmental disorders and epilepsy (collectively, termed SNAREopathies^9^). In our model system, we did not observe major changes in evoked FM dye unloading. These latter effects may reflect some compensation from other cooperative proteins known to function together to chaperone SNARE assembly, including MUNC13-1.^24,25^ The experimental paradigm used (elevated extracellular potassium) is also quite extreme, and while this has been used in many previous studies, it is possible that a more physiological assay may yet show an effect in STXBP1 knock-down conditions.^26–28^

In summary, we present a new experimental approach for understanding human cortical development at a stage approximately equivalent to 14–18 pcw. Loss of STXBP1 at this stage has divergent effects on glutamatergic and GABAergic synapses in the subplate, reducing the former, but increasing the latter. The synaptic networks in this region are proposed to be important for guiding neurogenesis, neuron migration and formation of thalamocortical and cortico-cortical connectivity.^4,29,30^ Therefore, alterations in subplate synaptic activity are expected to have a large effect on the subsequent development of the forebrain. Treatment of STXBP1-related disorders is challenging, requiring a personalized treatment plan.^10^ This model system could serve as a crucial bridge to facilitate the development of future precision-medicine approaches.

## Supplementary Material

awac396_Supplementary_Data

## Supplementary material

Supplemementary material is available at *Brain* online.
