# Supplementary material for: Altered synaptic connectivity in an in vitro human model of STXBP1 encephalopathy
Source: Brain. 2022 Oct 31;146(3):850–7. doi: 10.1093/brain/awac396 (PMC9976961; doi:10.1093/brain/awac396)
Supplement: awac396_Supplementary_Data [file awac396_supplementary_data.pdf]

Supplementary Material:

Altered synaptic connectivity in an *in vitro* human model of STXBP1 encephalopathy

Faye McLeod, Anna Dimtsi, Amy C Marshall, David Lewis-Smith, Rhys Thomas, Gavin J Clowry and Andrew J Trevelyan

- Supplementary Methods
- References
- Supplementary Tables
    - Supplementary Table 1
    - Supplementary Table 2

- Supplementary Figures
    - Supplementary Fig. 1
    - Supplementary Fig. 2
    - Supplementary Fig. 3
    - Supplementary Fig. 4

Supplementary Methods

Human organotypic slice cultures

Tissue was transferred to oxygenated ice-cold high sucrose ACSF containing (in mM): 180 sucrose, 25 $NaHCO_3$, 10 glucose, 10 $MgSO_4$, 2.5 KCl, 2 N-acetylcysteine, 1.25 NaH_2PO_4, 1 taurine, 1 ascorbic acid, 0.5 $CaCl_2$, 0.1 aminoguanidine hydrochloride, 0.044 indomethacin, 0.044 ethyl pyruvate and adjusted to 300–310 mOsM and 7.4 pH. Subsequently 280µm slices were cut using a 5100mz Vibratome (Camden Instruments). Cortical slices were placed inside 6-transwell plates containing BrainPhys (5790; Stemcell Technologies) culture media supplemented with 1 x N2, 1 x B27, 40ng/ml Brain-derived Neutrophic Factor (BDNF), 20ng/ml Glia-derived Neurotrophic Factor (GDNF), 30ng/ml Wnt7a, 200 nM ascorbic acid, 1 mM dibutyryl cyclic AMP and 1 µg/ml laminin.

Live imaging

Live imaging was performed using an inverted Zeiss LSM800 Airyscan confocal microscope. AAV9-CMV-GFP infected slices were placed in culture media and maintained at 37°C, 5% CO_2. Images were acquired in the subplate using a x20 objective (Numerical Aperture; NA = 0.8) every 30 seconds for 15 minutes from the same slice for 4 weeks generating final image resolutions of 512 x 512 pixels.

FM4-64 dye was utilised as previously described[1]. Briefly, infected slices were transferred to control solution containing (in mM, pH 7.3): 120 NaCl, 10 HEPES, 10 Glucose, 2 $CaCl_2$ and 1 $MgCl_2$ to equilibrate. After 5 minutes, they were placed in control solution, FM4-64 dye (7.5 µM) and potassium chloride (KCl; 50 mM with osmolarity compensated) for 90 seconds to load the dye into vesicles. Subsequently, slices were placed for 60 seconds in control solution and FM4-64 dye. Finally, slices were transferred to control solution to wash off any excess non-internalised dye for at least 10 minutes.

Spontaneous exocytosis (non-evoked FM4-64 dye unloading) was assessed by perfusing slices with control solution and consecutive images of GFP-positive axons and FM4-64

loaded vesicles were acquired every 3 seconds using a 40x objective (NA = 1.3) at room temperature (RT). On separate slices, to analyse evoked exocytosis (evoked FM4-64 dye unloading), slices were initially perfused with control solution (~60 seconds) then switched to control solution substituted with 50mM KCl (~150 seconds) to stimulate the cells.

Analysis was performed using Fiji software (NIH, RRID:SCR_002285). Regions of interest (ROIs) representing the vesicles, were identified for each slice. One ROI on an area with no signal (representing the background) and one ROI on an area with a stable signal (to account for the FM4-64 bleaching) were selected. The ROIs remained the same throughout the videos and their mean fluorescent measurements were quantified. Mean background values were subtracted from the measured signal, and each value was divided by the mean stable signal value. For spontaneous exocytosis ROI values were normalised to the average of the first five frames. Evoked exocytosis values were normalised to the average ROI values of the first five frames or to the 5 frames immediately before KCl stimulation to calculate the fraction of loaded vesicle pool. A final averaging of ROI values every 9 seconds was then performed.

Whole cell-patch clamp recordings

Slices were transferred into the recording chamber of an upright Leica DMLFSA fluorescent microscope fitted with Micro Control Instruments micromanipulators and continuously perfused at 34°C with oxygenated ACSF containing (in mM): 126 NaCl, 3 KCl, 1.25 NaH_2PO_4, 24 $NaHCO_3$, 10 Glucose, 1.2 $CaCl_2$ and 1 $MgSO_4$. Cells were voltage-clamped in whole cell configuration using borosilicate glass patch electrodes (5–8 MΩ) filled with an intracellular solution containing (in mM): 125 K-methyl-SO_4, 10 HEPES, 2.5 Mg-ATP, 6 NaCl, 290 mOsM and pH 7.35. All data were collected using an Axopatch 200B amplifier, filtered (1 kHz) and digitised (10 kHz). Miniature currents were monitored and analysed using WinEDR and WinWCP software (http://spider.science.strath.ac.uk/sipbs/software_ses.htm).

Immunohistochemistry

Slices were fixed in 4% paraformaldehyde (PFA)/4% sucrose in phosphate buffered saline (PBS; 0.12 M, pH 7.4; PBS) for 20 minutes at room temperature (RT). Staining was performed using the protocol optimised specifically for acute slices[2]. In brief, slices were incubated in blocking solution (10% donkey serum, 1% Triton X-100 in PBS) for

~ 4-6 hours at RT on a vibrating shaker. Various primary antibodies at differing dilutions in blocking solution (Supplementary Table 2) were applied overnight at 4°C. Slices were subsequently incubated with fluorophore-bound secondary antibodies (1:500; Life Technologies) for 2-3 hours at RT on a vibrating shaker before DAPI (1:5000) applied and mounted on glass slides in Fluoromount-G (SouthernBiotech).

Image analysis

The number as well as the length of primary subplate neuron processes was measured using Fiji software. Synaptic counts and integrated density (area × mean intensity) measurements were conducted using Zen 3.1 software (Zeiss; RRID:SCR_013672). These were optimised with a range of filters and thresholds to specifically identify the ROI for pre- and post-synaptic markers. Settings for markers of interest were kept consistent throughout all replicates. A synapse was defined as the colocalisation of specific pre- and post-synaptic markers with a minimum of 2 pixels overlapping. The counts of individual and colocalised ROIs were normalised to the volume of each image then represented per 100 μm^3.

Western blot

Membranes were blocked then probed overnight at 4°C on a vibrating shaker with anti STXBP1 antibody (1:1000; Abcam) and anti β-actin (1:5000; Millipore, A1978). Appropriate species specific HRP-conjugated secondary antibodies were added (BIO-RAD) at RT. Visualisation of protein bands was performed using a GBOX-Chemi-XRQ gel system (Syngene).

References

1 Cousin, M. A. *Curr Protoc Neurosci* **Chapter 2**, Unit 2 6, (2008).
2 McLeod, F. *et al. J Vis Exp*, (2017).

Supplementary Tables

Supplementary Table 1: List of human cases

Case	Age (pcw)	Brain Region	Experiment (Figure number)
FT1	17	Occipital	1, 2
FT 2	17	Occipital	1, 2
FT 3	14	Occipital	1, 2
FT 4	16	Occipital	1, 2
FT 5	16	Occipital	1, 2
FT 6	14	Occipital	1, 2
FT 7	17	Occipital	1, 2, 3, 4
FT 8	16	Occipital	1, 2, 3, 4
FT 9	18	Occipital	-
FT 10	14	Occipital	1, 2
FT 11	13	Occipital	-
FT 12	16	Occipital	1, 2, 3, 4
FT 13	17	Occipital	3, 4
FT 14	14	Occipital	-
FT 15	17	Occipital	4

Supplementary Table 2: Primary antibodies used

Primary antibody to	Species	Dilution	Supplier	RRID number
Synapsin 1	Rabbit pycl	1:500	Millipore	AB_2200400
vGlut1	Guinea Pig pycl	1:2000	Millipore	AB_2301751
GAD67	Mouse mocl	1:1000	Millipore	AB_2278725
Homer 1	Chicken pycl	1:1000	Synaptic Systems	AB_2631222
vGAT	Rabbit pycl	1:1000	Synaptic Systems	AB_887869
Gephyrin	Guinea Pig mocl	1:500	Synaptic Systems	AB_2661777
NeuN	Mouse mocl	1:1000	Abcam	AB_10711040
STXBP1	Rabbit mocl	1:500	Abcam	AB_10861492
MAP2	Chicken pycl	1:2000	Abcam	AB_2138153
GFP	Rabbit pycl	1:5000	Abcam	AB_303395

Supplementary Figures

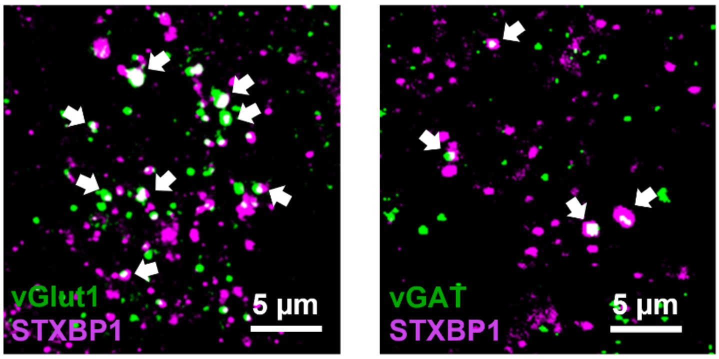

Supplementary Fig. 1: Localisation of STXBP1 to glutamatergic and GABAergic synaptic components. Example micrographs displaying pre-synaptic glutamatergic (vGlut1) and GABAergic (vGAT) components in the subplate colocalising with STXBP1 (arrows) at 17 pcw.

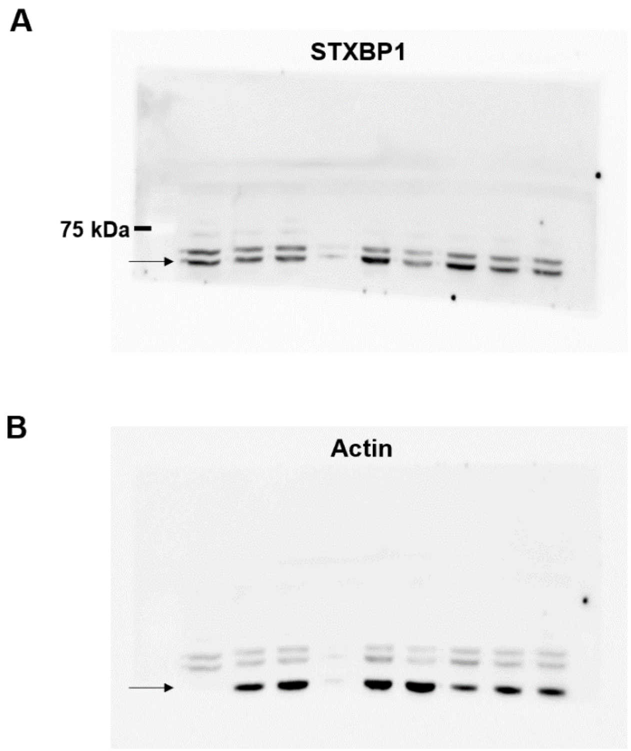

Supplementary Fig. 2: Full-length western blot membranes. (A) PVDF membrane displaying the STXBP1 bands (~68 kDa) used in Fig. 3A. Note the presence of two bands representing two isoforms of STXBP1. The bottom corresponds to the canonical sequence as it has migrated to approximately to 68 kDa (UniProt ID: P61764) and the top band likely corresponds with the known splice variant at approximately 69 kDa. **(B)** The same membrane was probed for β-Actin as a loading control (~42 kDa). Traces of the STXBP1 bands can be observed above the β-Actin as the membrane was not stripped. Arrows indicate the bands used for quantification.

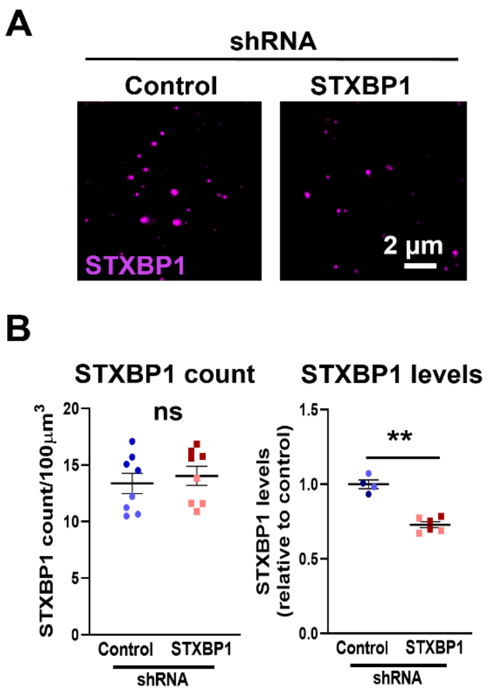

Supplementary Fig. 3: Genetic knock-down of STXBP1 in the subplate of human cortical slice cultures. (A) Photomicrographs of STXBP1 immunoreactivity in the subplate at 16 pcw following 2 weeks with scrambled control or *STXBP1* shRNA **(B)** Quantification of STXBP1 puncta count and integrated density levels in the subplate. Note the comparable terminal numbers (STXBP1 count) but less protein (STXBP1 levels). Analysis performed using an unpaired t-test, n = 4-8 slices/condition from 2 human samples). Data presented as scatter plots and mean ± SEM displaying all slice data points with each human sample per condition represented by a different blue/red colour shade.

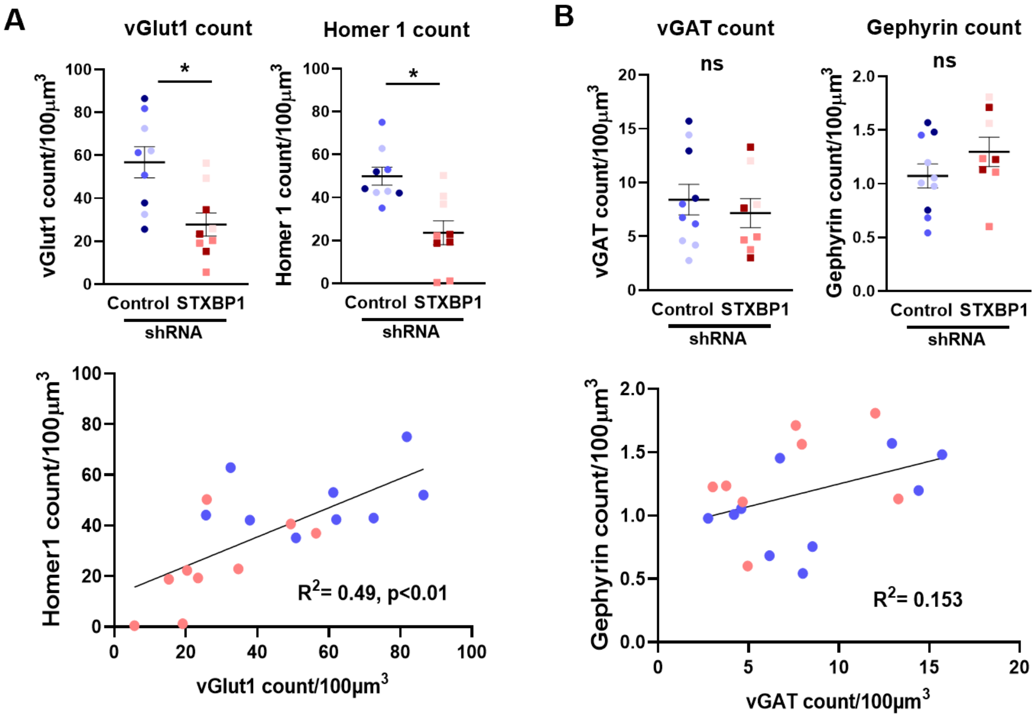

Supplementary Fig. 4: Altered pre- and post-synaptic components with loss of STXBP1. (A-B) Quantification of each pre- and post-synaptic component for glutamatergic synapses (A) and GABAergic synapses (B) in the subplate at 16 pcw following two weeks of scrambled control or *STXBP1* shRNA. Analysis performed using an unpaired t-test, n = 8-10 slices per condition from 3 human samples. Top panel data presented as scatter plots with mean ± SEM displaying all slice data points with each human sample per condition represented by a different blue/red colour shade. Bottom panel data represent the correlation found between pre- and post-synaptic counts (R^2 values indicated on each graph).
